# Antagonistic Regulation of ABA Responses by Duplicated Tandemly Repeated DUF538 Protein Genes in Arabidopsis

**DOI:** 10.3390/plants12162989

**Published:** 2023-08-19

**Authors:** Yingying Li, Wei Wang, Na Zhang, Yuxin Cheng, Saddam Hussain, Yating Wang, Hainan Tian, Hadia Hussain, Rao Lin, Yuan Yuan, Chen Wang, Tianya Wang, Shucai Wang

**Affiliations:** 1 Key Laboratory of Molecular Epigenetics of MOE, Northeast Normal University, Changchun 130024, China; liyy857@nenu.edu.cn (Y.L.); zhangn906@nenu.edu.cn (N.Z.); apple9036@163.com (Y.C.); wangyt814@nenu.edu.cn (Y.W.); tianhainan2012@126.com (H.T.); hadiahussain37@outlook.com (H.H.); linr944@nenu.edu.cn (R.L.); yuany759@nenu.edu.cn (Y.Y.); 15546477715@163.com (C.W.); wangty309@nenu.edu.cn (T.W.); 2 Laboratory of Plant Molecular Genetics & Crop Gene Editing, School of Life Sciences, Linyi University, Linyi 276000, China; wangwei220201@163.com (W.W.); botanistonline@yahoo.com (S.H.)

**Keywords:** ASDs, ABA, DUF538 proteins, CRISPR/Cas9, Arabidopsis

## Abstract

The plant hormone ABA (abscisic acid) regulates plant responses to abiotic stresses by regulating the expression of ABA response genes. However, the functions of a large portion of ABA response genes have remained unclear. We report in this study the identification of ASDs (ABA-inducible signal peptide-containing DUF538 proteins), a subgroup of DUF538 proteins with a signal peptide, as the regulators of plant responses to ABA in Arabidopsis. ASDs are encoded by four closely related DUF538 genes, with *ASD1*/*ASD2* and *ASD3*/*ASD4* being two pairs of duplicated tandemly repeated genes. The quantitative RT-PCR (qRT-PCR) results showed that the expression levels of *ASDs* increased significantly in response to ABA as well as NaCl and mannitol treatments, with the exception that the expression level of *ASD2* remained largely unchanged in response to NaCl treatment. The results of Arabidopsis protoplast transient transfection assays showed that ASDs were localized on the plasma membrane and in the cytosol and nucleus. When recruited to the promoter of the reporter gene via a fused GD domain, ASDs were able to slightly repress the expression of the co-transfected reporter gene. Seed germination and cotyledon greening assays showed that ABA sensitivity was increased in the transgenic plants that were over-expressing *ASD1* or *ASD3* but decreased in the transgenic plants that were over-expressing *ASD2* or *ASD4*. On the other hand, ABA sensitivity was increased in the CRISPR/Cas9 gene-edited *asd2* single mutants but decreased in the *asd3* single mutants. A transcriptome analysis showed that differentially expressed genes in the *35S:ASD2* transgenic plant seedlings were enriched in several different processes, including in plant growth and development, the secondary metabolism, and plant hormone signaling. In summary, our results show that *ASDs* are ABA response genes and that ASDs are involved in the regulation of plant responses to ABA in Arabidopsis; however, ASD1/ASD3 and ASD2/ASD4 have opposite functions.

## 1. Introduction

ABA (abscisic acid) is one of the classic plant hormones involved in the regulation of several different aspects of plant growth and development, such as seed germination, seedling development, and senescence [1,2]. Most importantly, ABA functions as a key hormone for regulating plant responses to abiotic stresses, including drought, heat, cold, and salinity [3,4,5,6,7,8,9,10].

ABA regulates plant responses to abiotic stresses via signal transduction [3,4,5,9,11,12]. ABA signaling starts from the binding of ABA molecules through the receptor proteins PYR1 (Pyrabactin resistance 1)/PYLs (PYR1-likes)/RCARs (regulatory component of ABA receptors) [7,13,14,15,16,17]. The binding of ABA enables the interaction of PYR1/PYLs/RCARs receptors with A-group PP2Cs (Protein phosphatase 2Cs) phosphatases, which are the negative regulators of ABA signaling that bind to the SnRK2s (sucrose nonfermenting 1 (SNF1)-related protein kinase 2s) kinases and inhibit their activities under normal conditions [5,9,12,18,19,20,21,22]. SnRK2s kinases, the positive regulators of ABA signaling, are self-activated once they are released from the A-group PP2Cs phosphatases, and they are then able to activate the downstream ABF (ABA-responsive element binding protein)/AREB (ABRE-binding factor)/ABI5 (ABA insensitive 5)-type bZIP (basic region leucine zipper) transcription factors [6,13,14,15,23]. Activated ABF/AREB/ABI5-type bZIP transcription factors, in turn, are capable of regulating the expression of ABA response genes and, thus, plant responses to abiotic stresses [9,10,11,19,20,21,22]. However, the functions of a large portion of ABA response genes have remained largely uncharacterized.

The DUF538 (domain of unknown function 538) proteins, a superfamily of proteins with poorly defined functions, are extensively distributed in Embryophyta [24,25]. A few pieces of available evidence indicate that DUF538 proteins are involved in the regulation of plant responses to environmental stresses. For example, the first full-length DUF538 cDNA was cloned from stress-challenged leaves of *Celosia cristata* plants [26]. DUF538 proteins have been predicted to be the functional equivalents of the BPI (bacterial permeability-increasing) proteins, a protein in the innate system of mammals that can bind to lipopolysaccharides on the outside leaflet of the bacteria, therefore hydrolyzing and eventually killing the bacteria [27]. Based on the predicated tertiary structures, DUF538 proteins may also be homologs of the esterase-type lipolytic enzymes [28]. Indeed, it has been shown that DUF538 proteins can act as chlorophyll-hydrolyzing enzymes to degrade chlorophyll molecules [25]; moreover, they can bind to pectin molecules [29], therefore regulating stress induced chlorophyll degradation and cell wall-associated defense responses in plants [25,29].

Furthermore, CaWSCP, a water-soluble chlorophyll-binding protein in *Chenopodium album*, was identified as a DUF538 protein [24], and an exogenous application of DUF538 proteins to tobacco (*Nicotiana tabacum*) elevated the activities of redox enzymes such as peroxidase, polyphenol oxidase, catalase, and phenylalanine ammonia lyase [30]. In addition, the DUF538 protein GLOSSY6 was found to play a role in the intracellular trafficking of cuticular waxes and drought stress tolerance in maize [31]. Recently, a DUF538 protein in poplar (*Populus tomentosa*) was found to modulate stomatal shape and density, therefore functioning as a component of the drought stress and adequate precipitation regulatory networks [32].

SVB (Smaller trichomes with variable branched) proteins, initially identified as a trichome morphology regulator, is the first functional characterized DUF538 protein in Arabidopsis [33,34]. SVB has also been reported as a PI(3)P and PI(3,5) P2 binding protein, [35] as well as a regulator of ER stress tolerance [34]. The characterization of double mutants between *SVB* and its homolog genes indicates that SVBs function redundantly to regulate trichome formation in Arabidopsis [36,37]. In addition, our previous results show that the expression of *SVBs* was regulated by ABA [36].

In this research, we found the expression of *ASDs* (*ABA*-*inducible signal peptide*-*containing DUF538 proteins*), a distinct subgroup of DUF538 protein genes that is also regulated by ABA, and we discovered that ASDs function as regulators of plant responses to ABA in Arabidopsis. We also found that members of ASDs have similar and different functions, i.e., ASD1/ASD3 negatively, but ASD2/ASD4 positively regulate the ABA response in Arabidopsis.

## 2. Results

### 2.1. ASDs in Arabidopsis

By using the strategy previously described to identify novel regulators of ABA and/or abiotic stress responses in Arabidopsis [9], we found that the expression levels of three DUF538 genes, i.e., *At1g02816* (*ASD2*), *At4g02360* (*ASD3*), and *At4g02370* (*ASD4*), were increased in response to ABA treatment. According to the transcriptome data, their FPKM values were 14.29/94.36, 1.64/25.93, and 54.46/114.84, respectively, in the control/ABA seedlings.

**Figure 1:**
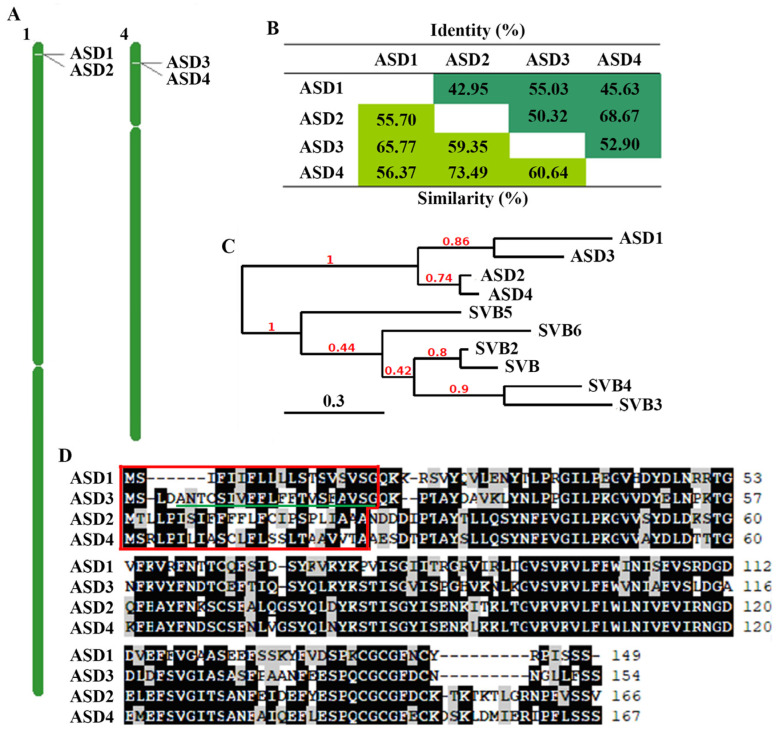
*ASDs* in Arabidopsis. (**A**) Distribution of *ASDs* on the chromosome. *ASD1* and *ASD2* are tandemly repeated genes on chromosome 1, and *ASD3* and *ASD4* are tandemly repeated genes on chromosome 4. (**B**) Amino acid identity and similarity of ASDs. The percentages of amino acid similarities and identities of ASDs were calculated by using the SIAS tool (http://imed.med.ucm.es/Tools/sias.html, accessed on 2 May 2023). The percentages of amino acid similarities were shaded in light green, and the percentages of identities in green. (**C**) Phylogenetic analysis of ASDs and SVBs. The full-length amino acid sequence of ASDs and SVBs were subjected to a phylogenetic analysis (https://www.phylogeny.fr/, accessed on 2 May 2023) by using the “one click” mode with default settings on Phylogeny. The number above the branch indicates the branch support values. The bar indicates branch length. (**D**) Amino acid sequence alignment of ASDs. Identical amino acids are shaded in dark and similar ones are shaded in gray; boxed sequences are signal peptides. Underline indicates the transmembrane domain of ASD3; the transmembrane domain was predicted by using TMHMM-2.0 (https://services.healthtech.dtu.dk/services/TMHMM-2.0/, accessed on 12 August 2023).

A bioinformatics analysis showed that *ASD3* and *ASD4* in addition to *ASD2* and another DUF538 gene *At1g02813* (*ASD1*) are tandemly repeated genes (Figure 1A); that ASDs shared high amino acid identities and similarities (Figure 1B); and that ASD1 is closely related to ASD3 while ASD2 is closely related to ASD4. Together, these genes formed a clade that was distinct to the SVBs clade (Figure 1C). In addition, in contrast to SVBs, which do not have signal peptides [36], ASDs have signal peptides (Figure 1D). ASD3 also contains a predicted transmembrane domain [37], but it is within the signal peptide of ASD3. Based on these characteristics, we named this subgroup of DUF538 protein genes collectively as *ABA*-*inducible signal peptide*-*containing DUF538 proteins* (*ASDs*).

### 2.2. ASDs Are ABA Response Genes

**Figure 2:**
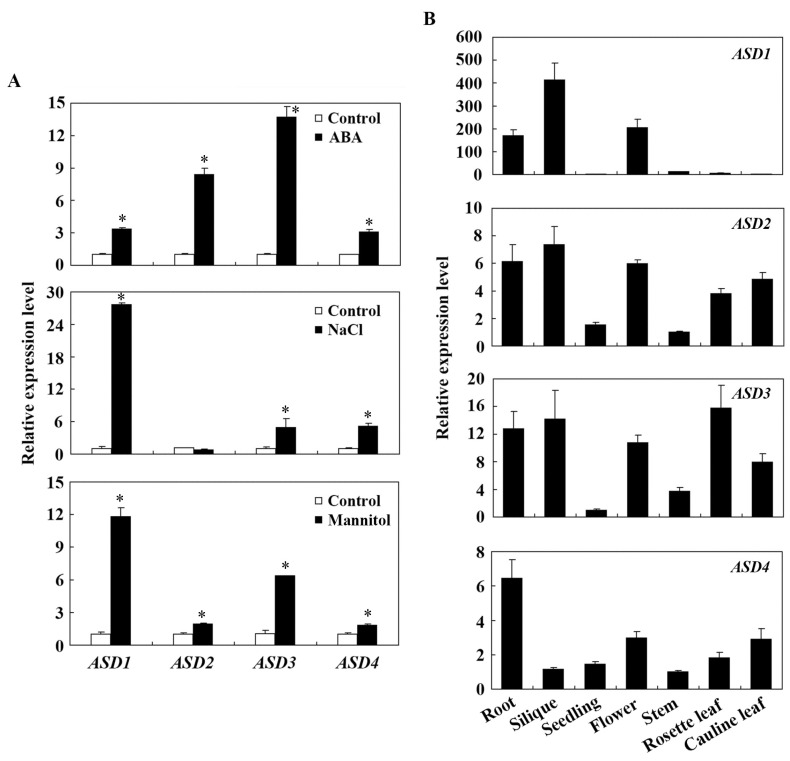
Expression of *ASDs* in response to ABA and abiotic stresses in different tissues and organs. (**A**) Expression of *ASDs* in response to ABA, NaCl, and mannitol treatments. Twelve-day-old seedlings of the Col wild-type that were grown in 1/2 MS plates were treated with 50 µM ABA, 150 mM Nacl, and 300 mM mannitol, respectively, or mock treated for 4 h in darkness on a shaker at 40 rpm. Total RNA was then isolated and used as a template for the quantitative RT-PCR (qRT-PCR) analysis. *ACT2* was used as an inner control for the qRT-PCR analysis, and the expression levels of *ASDs* in the mock-treated control seedlings were set as 1. The data represent the mean ± SD of three replicates; * Significantly different from the control (Student’s *t*-test, *p* < 0.005). (**B**) Expression patterns of *ASDs*. Twelve-day-old Col wild-type seedlings were grown in 1/2 MS, and the roots, siliques, flowers, stems, rosette leaves, and cauline leaves were collected from five-week-old soil grown Col wild-type plants; the total RNA was isolated for qRT-PCR analysis to examine the *ASDs*. *ACT2* was used as an inner control for the qRT-PCR analysis, and the data represent the mean ± SD of three replicates. The expression levels of *ASD1* in cauline leaves, *ASD2* in stems, *ASD3* in seedlings, and *ASD4* in stems were set as 1.

To examine if the expression of *ASDs* was indeed inducible by ABA, we compared the expression levels of *ASDs* in the control and ABA-treated seedlings. Seedlings of Col wild-type plants were treated with ABA or mock-treated with the solvent methanol as control for 4 h; RNA was isolated, and the expression levels of *ASDs* were then examined using quantitative RT-PCR (qRT-PCR). We found that the expression levels of all *ASDs*, including *ASD1*, increased in response to ABA treatment but to different levels, ranging from 3–14 folds (Figure 2A). Consistent with these findings, the bioinformatics analysis showed that the promoters of all four genes contain multiple ABA response elements (Figure S1).

Considering that the first full-length DUF538 cDNA was cloned from stress-challenged plants [26], we also examined the expression of *ASDs* in response to abiotic stresses, including salt and drought stresses. Seedlings of Col wild-type plants were treated with NaCl or mannitol for 4 h; the RNA was isolated, and the expression levels of *ASDs* were examined using a qRT-PCR analysis. As shown in Figure 2A, the expression levels of all *ASDs* but *ASD2* increased to NaCl treatment, and the expression levels of all *ASDs* increased to mannitol treatment.

We also examined the expression pattern of *ASDs* in Arabidopsis. Different tissues and organs were collected, the RNA was isolated, and the qRT-PCR analysis was used to examine the expression levels of *ASDs*. We found that all *ASDs* but *ASD1* were expressed in all of the tissues and organs examined and showed largely similar expression patterns, whereas *ASD1* was more specifically expressed in roots, siliques, and flowers (Figure 2B).

### 2.3. ASDs Are Associated with the Plasma Membrane but Are Also Located in the Cytosol and Nucleus

**Figure 3:**
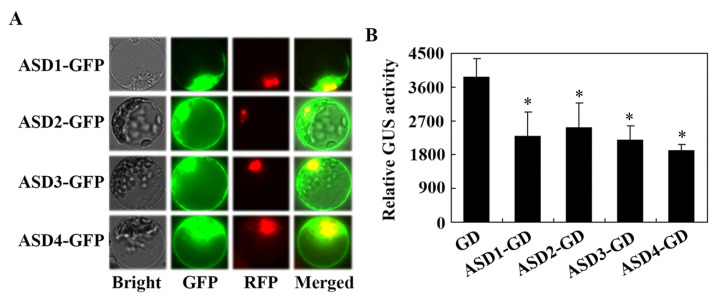
Sub-cellular localization and transcriptional activities of ASDs. (**A**) Sub-cellular localization of ASDs. The plasmids of *ASD1*-*GFP*, *ASD2*-*GFP*, *ASD3*-*GFP*, and *ASD4*-*GFP* were co-transfected, respectively, with the nucleus indicator *NLS*-*RFP* into isolated Arabidopsis protoplasts. The transfected protoplasts were incubated at room temperature in darkness for 20–22 h; the GFP and RFP fluorescence were examined and photographed under a confocal microscope (Olympus, Tokyo, Japan). (**B**) Transcriptional activities of ASDs. The plasmids of *ASD1*-*GD*, *ASD2*-*GD*, *ASD3*-*GD*, and *ASD4*-*GD* were co-transfected, respectively, with the *LD*-*VP* activator gene and the *LexA*-*Gal4: GUS* reporter gene into isolated Arabidopsis protoplasts. The transfected protoplasts were incubated at room temperature in darkness for 20–22 h, and the GUS activities were assayed using a Synergy HT fluorescence microplate reader (BioTEK, Winooski, VT, USA). The co-transfection of GD plasmids was used as a control. The data represent the mean ± SD of three replicates. * Significantly different from the GD (Student’s *t*-test, *p* < 0.05).

Previously, we showed that SVBs, members of the Arabidopsis DUF538 proteins, have different patterns in the sub-cellular localization; SVB, SVB2, SVB3, and SVB5 were observed all over the cells, but SVB4 and SVB6 were mainly localized in the nucleus [36]. Considering that ASDs are also DUF538 proteins but form a subgroup that is distinct to SVBs (Figure 1C) and that protein functions are related to the sub-cellular localization, we therefore examined the sub-cellular localization of ASDs by using Arabidopsis protoplast transfection assays to determine whether ASDs and SVBs may have different sub-cellular localization patterns. Considering that ASDs contain signal peptides (Figure 1D), we generated *ASDs* constructs with a C-terminal fused *GFP*. Plasmids of *ASDs*–*GFP* construct genes were co-transfected with *NLS*-*RFP*, a nucleus indicator gene, into Arabidopsis protoplasts. The transfected protoplasts were incubated overnight in darkness, and then the GFP and RFP fluorescence in the protoplasts was observed under a confocal microscope. As shown in Figure 3A, GFP fluorescence was observed overall in all of the cells, with strong fluorescence being observed on the cell membrane as well as in the cytosol and nucleus.

As an accumulation of ASDs was observed in the nucleus and because DUF538 proteins have been shown to be a component of the stress-related regulatory network [32], we thus examined whether ASDs may have transcriptional activities in the transfected protoplast. Plasmids of the *ASDs*-*GD* effect genes were co-transfected with the *LexA*-*Gal4:GUS* reporter gene and the *LD*-*VP* transcription activator gene into Arabidopsis protoplasts, and GUS activities were measured by using a microplate reader after the transfected protoplasts were incubated overnight in darkness. We found that, when compared with the co-transfected *GD* control gene, GUS activities in protoplasts co-transfected with the *ASDs*-*GD* genes were reduced, even though it was only in the amount of ~50% (Figure 3B), indicating that ASDs are able to repress reporter gene expression when being recruited to the promoter region of the reporter gene through a fused DNA binding domain.

### 2.4. ABA Sensitivity Is Increased in Transgenic Plants Over-Expressing ASD1/ASD3 but Decreased in Transgenic Plants Over-Expressing ASD2/ASD4

To examine if ASDs are involved in the regulation of plant responses to ABA in Arabidopsis, we generated transgenic plants that were over-expressing *ASDs* (Figure S2) and compared the ABA responses in the *ASD* over-expression transgenic plants with Col wild-type plants.

**Figure 4:**
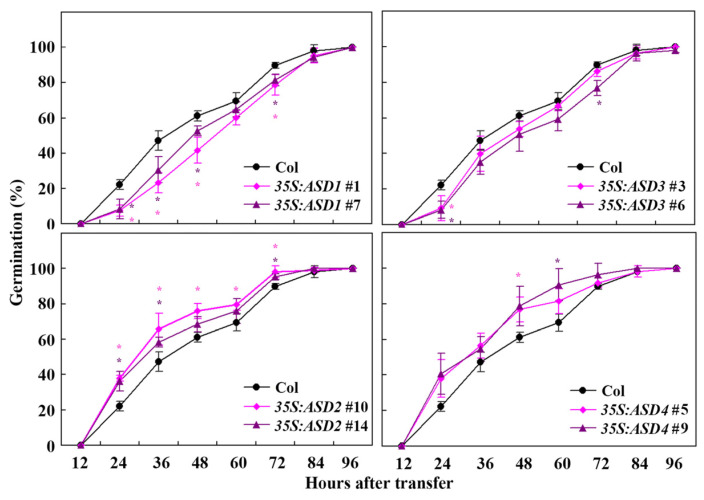
Effects of ABA on seed germination of Col wild-type and *35S:ASD* transgenic plants. Sterilized seeds were sown on 1/2 MS plates with or without 1 µM of ABA; the plates were kept at 4 °C in darkness for 2 days and then transferred to a growth room. The numbers of germinated seeds were counted every 12 h after the transfer, and the percentage of germination rate was calculated. The data represent the mean ± SD of three replicates. * Significantly different from the Col wild-type (Student’s *t*-test, *p* < 0.05). Please note that all data in this figure were from one representative experiment, but the data of the transgenic plants for each of the *ASDs* genes were plotted separately with data of the Col wild-type in order to clearly observe the differences.

Two different transgenic lines for each of the *ASDs* were used to examine their ABA responses in seed germination and seedling greening assays. In the seed germination assays, we found that all seeds on the control plates geminated 36 h after the transfer (Figure S3). On the ABA-treated plates, reduced germination rates were observed for seeds of the transgenic plants that were over-expressing *ASD1* or *ASD3* when compared with the seeds of the Col wild-type plants (Figure 4). On the other hand, increased germination rates were observed for seeds of the transgenic plants that were over-expressing *ASD2* or *ASD4* (Figure 4).

**Figure 5:**
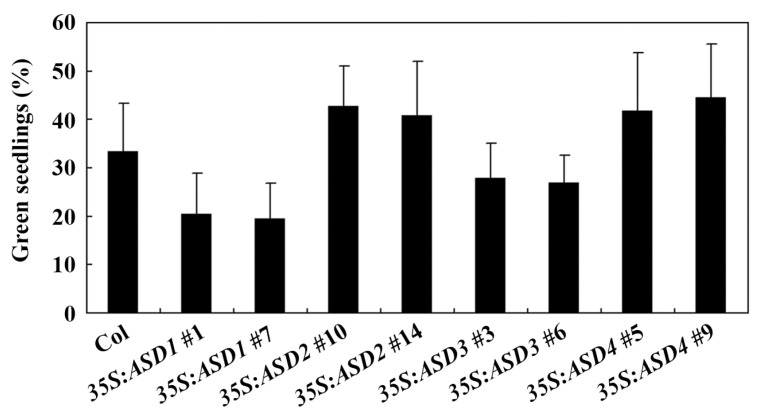
Effects of ABA on cotyledon greening of Col wild-type and *35S:ASD* transgenic plants. Sterilized seeds were sown on 1/2 MS plates with or without 1 µM of ABA; the plates were kept at 4 °C for 2 days under darkness and then transferred to a growth room, and green seedlings were counted 14 days after the transfer. The data represent the mean ± SD of three replicates.

In the seedling greening assays, similar results were obtained, i.e., ABA sensitivity was increased in transgenic plants over-expressing *ASD1* or *ASD3* but decreased in the transgenic plants over-expressing *ASD2* or *ASD4*, even though the differences are relatively mild (Figure 5). These results suggest that ASDs are involved in the regulation of plant sensitivity to ABA in Arabidopsis but that ASD1/ASD3 and ASD2/ASD4 have opposite functions.

### 2.5. ABA Sensitivity Is Increased in the asd2 Mutants but Decreased in the asd3 Mutants

As the data in the ABA sensitivity assays using transgenic over-expressed plants indicated that ASD1 and ASD3 have similar functions; that ASD2 and ASD4 have similar functions in regulating ABA response in Arabidopsis; and that ASD1/ASD3 and ASD2/ASD4 have opposite functions (Figure 4 and Figure 5), we decided to generate gene-edited mutants for *ASD2* and *ASD3* to further examine the roles of ASDs in regulating ABA response in Arabidopsis. Two differences targeting the sequences of *ASD2* and *ASD3*, respectively, were selected and used to generate gene-edited mutants using the CRISPR/Cas9 gene-editing technique.

**Figure 6:**
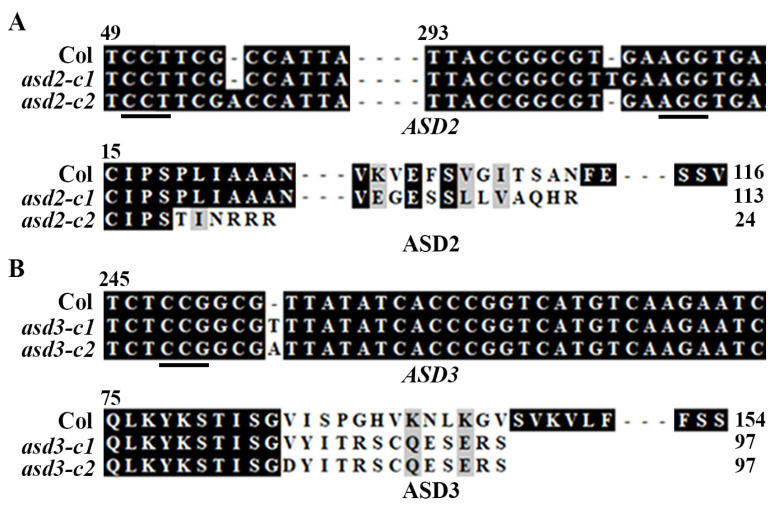
Generation of gene-edited mutants for *ASD2* and *ASD3*. (**A**) Alignment of nucleotide sequences of *ASD2* (upper panel) and amino acid sequences of ASD2 (lower panel) in Col wild-type and *asd2* mutant plants. For the nucleotide sequence alignment, the numbers indicate the relative position of the nucleotides to the start codon. For the amino acid sequences alignment, the ORF of *ASD2* in the mutants was identified by using ORF finder (www.ncbi.nlm.nih.gov/orffinder/, accessed on 15 September 2021), and the corresponding amino acid sequences were used for alignment with the amino acid sequence of the wild-type ASD2. (**B**) Alignment of nucleotide sequences of *ASD3* (upper panel) and amino acid sequences of ASD3 (lower panel) in Col wild-type and *asd3* mutant plants. For the nucleotide sequence alignment, the numbers indicate the relative position of the nucleotides to the start codon. For the amino acid sequences alignment, the ORF of *ASD3* in the mutants was identified by using ORF finder, and the corresponding amino acid sequences were used for alignment with the amino acid sequence of the wild-type ASD3.

Two different mutants were obtained for *ASD2* and *ASD3*, respectively. As shown in Figure 6A, in the *asd2*-*c1* mutant, the second target was edited and a single nucleotide was inserted; meanwhile, in the *asd2*-*c2* mutant, the first target was edited and a single nucleotide was inserted. In both of the *asd2* mutants, the nucleotide insertions led to a few amino acid substitutions and a premature stop in ASD2 (Figure 6A). In both the *asd3*-*c1* and *asd3*-*c2* mutants, the second target was edited and a single nucleotide was inserted, which led to a few amino acid substitutions and a premature stop in ASD3 (Figure 6B).

**Figure 7:**
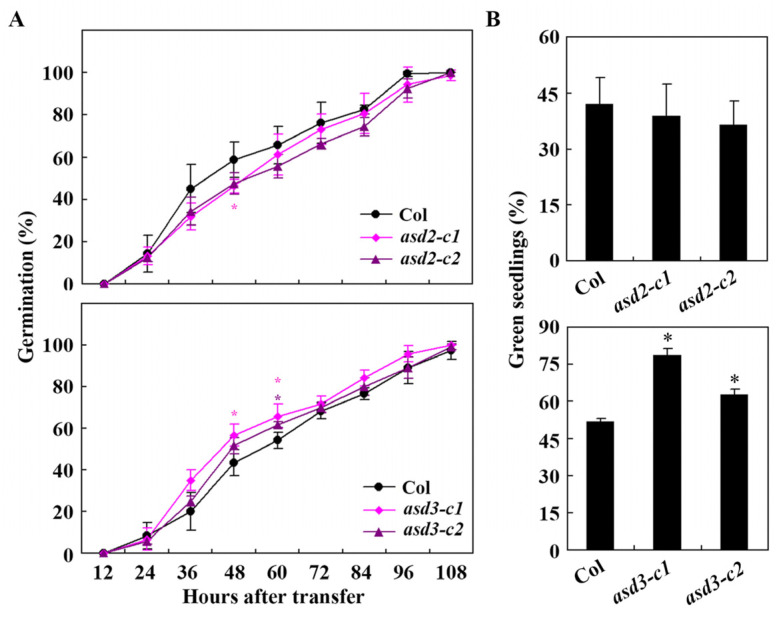
Effects of ABA on seed germination and cotyledon greening of Col wild-type plants and *asd2* and *asd3* mutants. (**A**) Effects of ABA on seed germination of Col wild-type plants and *asd2* and *asd3* mutants. Sterilized seeds were sown on 1/2 MS plates with or without 1 µM of ABA; the plates were kept at 4 °C in darkness for 2 days and then transferred to a growth room. The numbers of germinated seeds were counted every 12 h after the transfer, and the percentage of germination rate was calculated. The data represent the mean ± SD of three replicates. * Significantly different from the Col wild-type (Student’s *t*-test, *p* < 0.05). (**B**) Effects of ABA on cotyledon greening of Col wild-type plants and *asd2* and *asd3* mutants. Sterilized seeds were sown on 1/2 MS plates with or without 1 µM of ABA; the plates were kept at 4 °C in darkness for 2 days and then transferred to a growth room. The green seedlings were counted 14 days after the transfer. The data represent the mean ± SD of three replicates. * Significantly different from the Col wild-type (Student’s *t*-test, *p* < 0.01).

The *asd2* and *asd3* mutants generated were used to examine plant responses to ABA in seed germination and seedling greening assays. The results of the seed germination assays show that ABA sensitivity was increased in the *asd2* mutants but decreased in the *asd3* mutants (Figure 7A). In the seedling greening assays, ABA sensitivity in the *asd2* mutants slightly increased but clearly decreased in the *asd3* mutants (Figure 7B). These results further confirmed that ASD2 negatively, but ASD3 positively regulates ABA responses in Arabidopsis.

We also directly compared seed germination of the Col wild-type, the *35S:ASD2* and *35S:ASD3* transgenic plants, and the *asd2* and *asd3* mutants in response to a higher ABA concentration, i.e., 2 µM. The results were similar to that obtained with 1 µM, i.e., ABA sensitivity was increased in the *asd2* mutants and *35S:ASD3* transgenic plants but decreased in the *35S:ASD2* transgenic plants and *asd3* mutants (Figure S4). In addition, we performed ABA-inhibited root elongation assays by using the Col wild-type, the *35S:ASD2* and *35S:ASD3* transgenic plants, and the *asd2* and *asd3* mutants; no differences were observed in these plants (Figure S5). However, we noticed that the seedlings of the *35S:ASD2* transgenic plants produced longer, whereas the *asd2* mutants produced shorter primary roots (Figure S5).

### 2.6. ASD2 Affected Genes Are Enriched in Different Processes, including Plant Hormone Signaling

To examine how ASDs may regulate ABA responses in Arabidopsis, we wanted to identify genes that may be affected by ASDs. Considering that ASD1 and ASD3, as well as ASD2 and ASD4, may have redundant functions and that ABA sensitivities were decreased in transgenic plants overexpressing *ASD2*/*ASD4*, we decided to take ASD2 as an example for identifying the ASDs-regulated genes by comparing gene expression in seedlings of the *35S:ASD2* transgenic and Col wild-type plants at the transcriptome level using a transcriptome sequencing analysis. A total of 396 differentially expressed genes (DEGs) were identified, with 141 down-regulated and 255 up-regulated genes occurring in the *35S:ASD2* transgenic plants compared with the Col wild-type plants (Table S1). It is worth noticing that about 1/3 of the DEGs, i.e., 50 of the 141 down-regulated and 106 of the 255 up-regulated genes in the *35S:ASD2* transgenic plants, were ABA response genes (Table S2).

**Figure 8:**
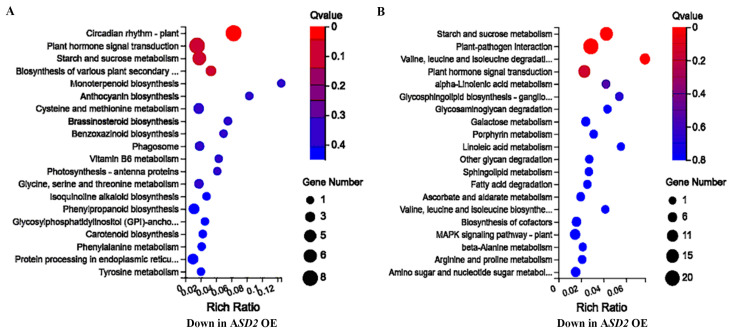
KEGG enrichment of the DEGs in the *35S:ASD2* transgenic plants. (**A**) KEGG enrichment of the DEGs that are down-regulated in the *35S:ASD2* transgenic plants. The graph presents the 20 KEGG pathways with the highest transcriptional variations out of the down-regulated DEGs in the *35S:ASD2* transgenic plants. (**B**) KEGG enrichment of the DEGs that are up-regulated in the *35S:ASD2* transgenic plants. The graph presents 20 KEGG pathways with the highest transcriptional variations out of the up-regulated DEGs in the *35S:ASD2* transgenic plants.

A gene ontology (GO) analysis showed that the DEGs that were down-regulated in the *35S:ASD2* transgenic plants were mainly enriched in processes that included circadian rhythm, plant hormone signal transduction, and different metabolism and biosynthesis processes (Figure 8A). Similarly, the DEGs that were up-regulated in the *35S:ASD2* transgenic plants were also enriched in plant hormone signal transduction as well as metabolism and biosynthesis processes, but some of the processes were different from the down-regulated DEGs in the *35S:ASD2* transgenic plants (Figure 8B). In addition, the DEGs up-regulated in the *35S:ASD2* transgenic plants were also enriched in other processes such as plant–pathogen interactions and the MAPK signaling pathway (Figure 8B).

## 3. Discussion

DUF538 proteins are extensively distributed in Embryophyta [24,25], and very limited studies have indicated that DUF538 proteins are involved in regulating plant responses to environmental stresses [26,30,31,32]. Meanwhile, the plant hormone ABA plays a key role in regulating plant responses to abiotic stresses such as drought, heat, cold, and salinity [3,5]. We provide evidence from this study that ASDs, a subgroup of DUF538 proteins in Arabidopsis, are involved in the regulation of plant responses to ABA and may also be involved in the regulation of plant responses to abiotic stresses.

Firstly, we found that the expression levels of *ASDs* increased in response to the ABA treatment (Figure 2), suggesting that *ASDs* are ABA response genes. Secondly, we found that ABA sensitivity in the transgenic plants that were over-expressing *ASDs* were altered in both the seed germination and seedling greening assays (Figure 4 and Figure 5). In addition, altered ABA sensitivity was also observed in both the *asd2* and *asd3* mutants (Figure 7), and ABA sensitivity was altered in an opposite way to the transgenic plants over-expressing *ASDs* (Figure 4, Figure 5 and Figure 7). Thirdly, the expression levels of *ASDs* increased in response to abiotic stresses, including salt and drought (Figure 2). These results indicate that ASDs are involved in the regulation of plant responses to ABA in Arabidopsis and may also be involved in the regulation of plant responses to abiotic stresses.

Previously, we have shown that the expression levels of *SVBs*, another subgroup of DUF538 proteins genes, were increased in response to ABA treatment [36] and that SVBs function redundantly to regulate trichome formation in Arabidopsis [36,37]. In this study, we found that *ASDs* are also ABA response genes (Figure 2). However, in contrast to SVBs, ASDs have signaling peptides (Figure 1) and formed another subgroup that was distinct from SVBs (Figure 1). Most interestingly, we found that even though ASDs are closely related DUF538 proteins, ASD1/ASD3 and ASD2/ASD4 have opposite functions in regulating plant responses to ABA, i.e., ASD1/ASD3 negatively regulates responses but ASD2/ASD4 positively regulates plant responses to ABA (Figure 4, Figure 5 and Figure 7). Furthermore, *ASD1* and *ASD2* are tandemly repeated genes, so do *ASD3* and *ASD4*, but ASD1 is closely related to ASD3, and ASD2 is closely related to ASD4 (Figure 1). Thus, it is very likely that *ASD1*/*ASD2* and *ASD3*/*ASD4* are duplicated genes. Because both ASD1 and ASD3 negatively regulate plant responses; ASD2 and ASD4 positively regulate plant responses to ABA; and because the phenotypes of the single mutants are mild, it is very likely that ASD1 and ASD3, as well as ASD2 and ASD4, may have redundant functions. It will be of interest to examine whether this is indeed the case by generating double mutants; it will be also of interest to examine whether ASD1/ASD3 and ASD2/ASD4 function in same pathway to regulate plant responses to ABA by generating high-order mutants of *ASDs*.

On the other hand, even though ASD1/ASD3 and ASD2/ASD4 have opposite functions in regulating plant responses to ABA (Figure 4, Figure 5 and Figure 7), ASDs shared high amino acid identities and similarities (Figure 1). It is possible that some of the regions with low amino acid identities and similarities in the proteins, or/and the extra 8 or 9 amino acids presented in ASD2/4, are responsible for their different functions; it will be of great interest to examine if that is indeed the case by conducting domain-swapping experiments.

Considering that the previous results have shown that DUF538 proteins are involved in the regulation of plant responses to environmental stresses [26,30,31,32], the expression of *ASDs* were induced by abiotic stress treatments (Figure 2) and both up- and down-regulated DEGs in the *35S:ASD2* transgenic plants were enriched in plant hormone signal transduction (Figure 8). It is very likely that ASDs may also be involved in the regulation of plant responses to abiotic stresses. It is worthwhile to examine if this is the case and if ASD1 and ASD3, as well as ASD2 and ASD4, have redundant functions; it is also worth examining whether ASD1/ASD3 and ASD2/ASD4 function in the same pathway to regulate plant responses to abiotic stresses.

## 4. Materials and Methods

### 4.1. Plant Materials and Growth Conditions

Arabidopsis (*Arabidopsis thaliana*) of the Columbia-0 (Col) ecotype were used as the wild-type for protoplast isolation and plant transformation to generate transgenic over-expressed plants and gene-edited mutants, to examine the expression of *ASDs* to ABA treatment, and to serve as a control for plant ABA response assays and the transcriptome sequencing analysis.

For protoplast isolation and plant transformation, the Col wild-type seeds were soaked with sterile water and kept at 4 °C in darkness for 2 days; then, they were directly sown into soil pots and grown in a growth room.

For the RNA isolation, transcriptome sequencing analysis, and seed germination and seedling greening assays, seeds of the Col wild-type, *35S:ASDs* transgenic plants, and *asd2* and *asd3* single mutants were surface-sterilized for 10 min with 25% (*v*/*v*) bleach, washed with sterilized water four times, grown in 1/2 MS (Murashige and Skoog) plates with vitamins (Plant Media) and 1% (*w*/*v*) sucrose, and solidified with 0.6% (*w*/*v*) phytoagar (Plant Media). The plates were kept at 4 °C in darkness for 2 days and then transferred into a growth room.

The growth room was set to a long-day (16 h light/8 h dark) growth condition with a light density of ~120 µmol m^−^^2^ s *^−^*^1^ and a temperature of 22 °C.

### 4.2. Constructs

The reporter gene *LexA*-*Gal4:GUS*, control effector gene *GD*, and *NLS*-*RFP* nuclear indicator gene constructs have been previously reported [38,39,40].

To generate *GFP*- and *GD*-tagged *ASDs* constructs for sub-cellular localization and transcriptional activity assays, the full-length open reading frame (ORF) sequences of *ASDs* without the stop codon were RT-PCR amplified by using RNA that was isolated from 12-day-old seedlings of the Col wild-type, digested using proper enzymes and cloned in-frame with a C-terminal GFP tag and GD tag, respectively, into the *pUC19* vector under the control of the *CaMV 35S* promoter [41,42]. To generate *35S:ASDs* constructs for plant transformation, the full-length ORF sequence of *ASDs* were amplified, digested with proper enzymes, and cloned into the *pUC19* vector with an in-frame N-terminal HA tag while under the control of the *35S* promoter. The *pUC19*-*35S:ASDs* constructs were digested with proper enzymes and cloned into the binary vector *pPZP211* [43].

The primer used to amplify *ASD1* with the stop codon were 5′-CAACATATGTCCATTTTCATCATCTTCC-3′ and 5′-CAAGAGCTCTTAAGAGGAAGAGATAGGCCTATAA-3′, and the reverse primer used to amplify *ASD1* without the stop codon was 5′-CAAGAGCTCAGAGGAAGAGATAGGCCTATAA-3′. The primer used to amplify *ASD2* with the stop codon were 5′-CAACATATGACTCTGCTTCCGATTTC-3′ and 5′-CAAGAGCTCTTAAACCGAAGAAACAAAAGG-3′, and the reverse primer used to amplify *ASD2* without the stop codon was 5′-CAAGAGCTCAACCGAAGAAACAAAAGG-3′. The primer used to amplify *ASD3* with stop codon were 5′-CAACATATGTCGCTAGACGCGAA-3′ and 5′-CAAGAGCTCTCAAGAAGAAAATAAAAGCCC-3′, and the reverse primer used to amplify *ASD3* without stop codon was 5′-CAAGAGCTCAGAAGAAAATAAAAGCCC-3′. The primer used to amplify *ASD4* with the stop codon were 5′-CAACATATGTCTCGTCTCCCGATTT-3′ and 5′-CAAGAGCTCTCAAGACGAAGAAAGAAAAGG-3′, and the reverse primer used to amplify *ASD4* without the stop codon was 5′-CAAGAGCTCAGACGAAGAAAGAAAAGG-3′.

To generate the CRISPR/Cas9 construct for the gene editing of *ASD2* and *ASD3*, respectively, the exon sequences of *ASD2* and *ASD3* were used to identify target sequences on CRISPRscan (http://www.crisprscan.org/?page=sequence, accessed on 11 August 2019), and the potential off-target was evaluated using Cas-OFFinder (http://www.rgenome.net/cas-offinder/, accessed on 11 August 2019). Two specific target sequences were selected for each gene. The specific target sequences selected for editing *ASD2* were 5′-CGGCGGCGATTAATGGCGA (AGG) -3′ and 5′-ACGAAGCTTACCGGCGTGA (AGG) -3′, and the specific target sequences selected for editing *ASD3* were 5′- CTGTCTCTTTCGCCGTCTC(CGG) -3′ and 5′-TGACCGGGTGATATAACGC(CGG) -3′. The target sequences were cloned into the *pHEE*-*FT* vector following the previously described procedure [44]. The primers used to generate the *ASD2* gene-editing CRISPR/Cas9 construct were DT1-BsF (*ASD2*), 5′-ATATATGGTCTCGATTG CGGCGGCGATTAATGGCGAGTT -3′, DT1-F0 (*ASD2*), 5′-TGCGGCGGCGATTAATGGCGAGTTTTAGAGCTAGAAATAGC-3′, DT2-R0 (*ASD2*), 5′-AACTCACGCCGGTAAGCTTCGTCAATCTCTTAGTCGACTCTAC-3′, and DT2-BsR (*ASD2*), 5′-ATTATTGGTCTCGAAAC TCACGCCGGTAAGCTTCGTC-3′. The primers used to generate the *ASD3* gene-editing CRISPR/Cas9 construct were DT1-BsF (*ASD3*), 5′-ATATATGGTCTCGATTG CTGTCTCTTTCGCCGTCTCGTT-3′, DT1-F0 (*ASD3*), 5′-TGCTGTCTCTTTCGCCGTCTCGTTTTAGAGCTAGAAATAGC-3′, DT2-R0 (*ASD3*), 5′-AACGCGTTATATCACCCGGTCACAATCTCTTAGTCGACTCTAC-3′, and DT2-BsR (*ASD3*), 5′-ATTATTGGTCTCGAAACGCGTTATATCACCCGGTCAC-3′. The U626-IDF and U629-IDR primers used for the colony PCR and sequencing of the CRISPR/Cas9 constructs generated were performed as previously described [45].

### 4.3. Plant Transformation and Over-Expression in Transgenic Plants and Mutants Selection

Col wild-type Arabidopsis plants that were about 5 weeks old and that had several mature flowers on the main inflorescence stems were transformed via *GV3101* Agrobacterium to generate over-expressed plants and gene-edited *asd2* and *asd3* single mutants using the floral dip method [46].

To select T1 transgenic plants, T1 seeds were sown on 1/2 MS plates containing 100 µg/mL of carbenicillin and 50 µg/mL of kanamycin, and T1 transgenic plants were transferred into soil pots.

To isolate *35S:ASDs* transgenic over-expressed plants, T2 seeds were collected from T1 transgenic plants and sown on 1/2 MS plates containing 25 µg/mL of kanamycin to select lines with 3:1 segregation. T3 seeds were collected and sown on 1/2 MS plates containing 25 µg/mL of kanamycin to identify homozygous lines. The homozygous lines of *35S:ASDs* transgenic plants with high expression levels were selected and used for the experiments.

To isolate the *asd2* and *asd3* gene-edited transgene-free mutants, the gene editing status in T1 plants with early flowering phenotypes was examined using a PCR amplification and sequencing of the genomic sequences of *ASD2* and *ASD3*, respectively. T2 seeds from gene-edited T1 plants were sown in soil pots, and the gene editing status of normal flowering T2 plants were examined using a PCR amplification and sequencing of the genomic sequences of *ASD2* and *ASD3*, respectively. The *Cas9*-free homozygous mutants were used for the experiments.

### 4.4. DNA Isolation and PCR

To examine the gene-editing status of *ASD2* and *ASD3*, the leaves of the T1 transgenic plants or *Cas9*-free T2 plants were used for DNA that was isolated by the SDS method and used for the PCR amplification of the genomic sequences of *ASD2* and *ASD3*. The PCR products were sequenced, and the sequencing results were examined and aligned with wild-type genome sequences of *ASD2* and *ASD3*. To identify the *Cas9*-free mutants, the DNA was isolated from leaves of the T2 plants and used for the PCR amplification of the *Cas9* fragment using the previously described primers [47].

### 4.5. Plasmid DNA Isolation, Protoplasts Isolation, and Transfection

Plasmid DNA including *ASDs*-*GFP*, *NLS*-*RFP*, *GD*, *ASDs*- *GD*, *LD*-*VP*, and *LexA*-*Gal4:GUS* were isolated by using a GoldHi EndoFree PlasmidMaxi Kit (CWBIO, Taizhou, China) according to the manufacturers’ protocols provided with the kit. Protoplasts were isolated from rosette leaves collected from 3–4-week-old Col wild-type plants, as previously described [9,42,48].

For the sub-cellular localization assay, plasmids of *ASDs*-*GFP* and *NLS*-*RFP* were co-transfected into the protoplasts. For transcriptional activity assays, *LexA*-*Gal4:GUS*, *LD*-*VP* and *ASDs*-*GD* or *GD* were co-transfected into Arabidopsis protoplasts. The transfected protoplasts were incubated under darkness at room temperature for 20–22 h; GFP and RFP florescence were examined and photographed under a confocal microscope (Olympus, Tokyo, Japan), and GUS activities were assayed using a Synergy HT fluorescence microplate reader (BioTEK).

### 4.6. RNA Isolation and Quantitative RT-PCR (qRT-PCR)

To examine the expression of *ASDs* in response to ABA, NaCl, and mannitol treatments, 12-day-old seedlings of the Col wild-type that were grown in 1/2 MS plates were treated with 50 µM of ABA, 150 mM of NaCl, and 300 mM of mannitol, respectively, or mock-treated for 4 h in darkness on a shaker at 40 rpm; then, the total RNA was isolated. To examine the expression pattern of *ASDs*, 12-day-old seedlings of the Col wild-type were grown in 1/2 MS, and the roots, siliques, flowers, stems, rosette leaves, and cauline leaves from 5-week-old soil grown Col wild-type plants were collected; then, the total RNA was isolated.

The total RNA was isolated and the cDNA was synthesized as previously described [9]. Briefly, the EasyPure Plant RNA Kit (TransGen Biotech, Beijing, China) was used for the total RNA isolated, and the cDNA was synthesized by using the EasyScript First Strand DNA Synthesis Super Mix (TransGen Biotech, Beijing, China) according to the protocols provided by the manufacturer. The primers used for the qRT-PCR analysis to examine the expression of *ASD1* were 5′-AAGGTGTCCACGACTACGAC-3′ and 5′-GATGCCGGAGATTACGGGTT-3′, the primers used to examine the expression of *ASD2* were 5′-AGATCACGAAGCTTACCGGC-3′ and 5′-CACACTGTGGCGACTCGTAA-3′; the primers used to examine the expression of *ASD3* were 5′-TTACGACGCCGTCAAACTCT-3′ and 5′-TCCGACGGAGAAGTCGAGAT, and the primers used to examine the expression of *ASD4* were 5′-AACAGGCAAATTCCATGCGT-3′ and 5′-ACAACCACACTGAGGCGATT-3′. *ACTIN2* (*ACT2*) was used as an inner control for the qRT-PCR analysis, and primers for *ACT2* have been previously described [49].

### 4.7. Seed Germination and Cotyledon Greening Assays

For the seed germination and cotyledon greening assays, sterilized seeds (30–40 seeds for each genotype) were sown on ½ MS plates with or without 1 µM of ABA, where the plates were kept at 4 °C for 2 days under darkness and then transferred to a growth room. The numbers of germinated seeds were counted every 12 h after the transfer, the green seedlings were counted 14 days after the transfer, and the germination and green seedlings rate was calculated. Three to four replicates were used for each experiment, and the experiments were repeated at least three times with similar results.

### 4.8. Transcriptome Analysis

Seeds of Col wild-type and *35S:ASD2* #10 transgenic plants were surface-sterilized for 10 min with 25% (*v*/*v*) bleach, washed with sterilized water four times, and sown on 1/2 MS medium and kept at 4 °C for 2 days; then, they were transferred to the growth room. Ten-day-old seedlings were collected, frozen in liquid nitrogen, and sent to the Beijing Genomics Institute (Shenzhen, China) for the transcriptome analysis. Three replicates for each genotype were used for transcriptome sequencing.

The sequencing data was filtered to remove reads containing the sequencing adapter and a low-quality base ratio of more than 20% using SOAPnuke [50], and clean reads were obtained and stored in the FASTQ format. The cleaned reads were mapped onto the Arabidopsis reference genome TAIR10 using HISAT2 [51], and the differential expression analysis was performed using the DESeq2 package with a Q value ≤ 0.05. The KEGG (https://www.kegg.jp/, accessed on 29 April 2023) enrichment analysis of the annotated differently expressed gene was performed by Phyper (https://en.wikipedia.org/wiki/Hypergeometric_distribution, accessed on 29 April 2023) based on the hypergeometric test. The significant levels of terms and pathways were corrected by the Q value using a rigorous threshold (Q value ≤ 0.05) [52].

## 5. Conclusions

Our data in this study show that *ASDs* are ABA response genes and that ASDs are involved in the regulation of plant responses to ABA. Furthermore, ASD1 and ASD3, as well as ASD2 and ASD4, have redundant functions, but ASD1/ASD3 and ASD2/ASD4 have opposite functions for regulating plant responses to ABA.

## Data Availability

All data are presented in the manuscript and Supplementary Materials.

## Supplementary Materials

The following supporting information can be downloaded at: https://www.mdpi.com/article/10.3390/plants12162989/s1, Table S1: List of differentially expressed genes in the *35S:ASD2* over-expression transgenic plant. Table S2: List of ABA response genes in the DEGs in the *35S:ASD2* over-expression transgenic plant. Figure S1: ABA response elements (ABREs) in the 2000 bp promoter region of *ASDs*. Figure S2: Expression of *ASD* genes in the overexpression transgenic plants. Figure S3: Seed germination of the Col wild-type, *35S:ASD* transgenic plants, and single mutants *asd2* and *asd3* in the control plates, as indicated in Figure 4 and Figure 7A, respectively. Figure S4: Effects of ABA on seed germination in the Col wild-type, *35S:ASD2*, and *35S:ASD3* transgenic plants and the *asd2* and *asd3* mutants. Figure S5: Effects of ABA on root elongation of the Col wild-type, *35S:ASD2*, and *35S:ASD3* transgenic plants and the *asd2* and *asd3* mutants.
