# Supplementary material for: Antagonistic Regulation of ABA Responses by Duplicated Tandemly Repeated DUF538 Protein Genes in Arabidopsis
Source: Plants (Basel). 2023 Aug 19;12(16):2989. doi: 10.3390/plants12162989 (PMC10459309; doi:10.3390/plants12162989)
Supplement: Supplementary file 1 [file plants-12-02989-s001.zip › Supplemental Figures.pdf]

ASD1	ABRE	ACGTG	44	5	+	Arabidopsis thaliana	cis-acting element involved in the abscisic acid responsiveness
	ABRE	CACGTG	85	6	+	Arabidopsis thaliana	cis-acting element involved in the abscisic acid responsiveness
	ABRE	ACGTG	86	5	+	Arabidopsis thaliana	cis-acting element involved in the abscisic acid responsiveness
	ABRE	AACCCGG	198	7	-	Arabidopsis thaliana	cis-acting element involved in the abscisic acid responsiveness
	ABRE	AACCCGG	1523	7	-	Arabidopsis thaliana	cis-acting element involved in the abscisic acid responsiveness
	ABRE	CACGTG	1545	6	-	Arabidopsis thaliana	cis-acting element involved in the abscisic acid responsiveness
	ABRE	ACGTG	1546	5	+	Arabidopsis thaliana	cis-acting element involved in the abscisic acid responsiveness
	ABRE	CACGTG	1875	6	-	Arabidopsis thaliana	cis-acting element involved in the abscisic acid responsiveness
	ABRE	ACGTG	1876	5	+	Arabidopsis thaliana	cis-acting element involved in the abscisic acid responsiveness
ASD2	ABRE	AACCCGG	740	7	-	Arabidopsis thaliana	cis-acting element involved in the abscisic acid responsiveness
	ABRE	CACGTG	762	6	-	Arabidopsis thaliana	cis-acting element involved in the abscisic acid responsiveness
	ABRE	ACGTG	763	5	+	Arabidopsis thaliana	cis-acting element involved in the abscisic acid responsiveness
	ABRE	CACGTG	92	6	+	Arabidopsis thaliana	cis-acting element involved in the abscisic acid responsiveness
	ABRE	ACGTG	93	5	+	Arabidopsis thaliana	cis-acting element involved in the abscisic acid responsiveness
	ABRE	GACACGTGGC	492	9	-	Triticum aestivum	cis-acting element involved in the abscisic acid responsiveness
	ABRE	CACGTG	494	6	+	Arabidopsis thaliana	cis-acting element involved in the abscisic acid responsiveness
	ABRE	ACGTG	495	5	+	Arabidopsis thaliana	cis-acting element involved in the abscisic acid responsiveness
	ABRE	GACACGTGGC	947	9	-	Triticum aestivum	cis-acting element involved in the abscisic acid responsiveness
	ABRE	CACGTG	949	6	-	Arabidopsis thaliana	cis-acting element involved in the abscisic acid responsiveness
	ABRE	ACGTG	950	5	+	Arabidopsis thaliana	cis-acting element involved in the abscisic acid responsiveness
ASD3	ABRE	ACGTG	643	5	-	Arabidopsis thaliana	cis-acting element involved in the abscisic acid responsiveness
	ABRE	AACCCGG	1247	7	+	Arabidopsis thaliana	cis-acting element involved in the abscisic acid responsiveness
	ABRE	CACGTG	1823	6	-	Arabidopsis thaliana	cis-acting element involved in the abscisic acid responsiveness
	ABRE	ACGTG	1824	5	+	Arabidopsis thaliana	cis-acting element involved in the abscisic acid responsiveness
	ABRE	CACGTG	1850	6	-	Arabidopsis thaliana	cis-acting element involved in the abscisic acid responsiveness
	ABRE	ACGTG	1851	5	+	Arabidopsis thaliana	cis-acting element involved in the abscisic acid responsiveness
ASD4	ABRE	AACCCGG	51	7	+	Arabidopsis thaliana	cis-acting element involved in the abscisic acid responsiveness
	ABRE	CACGTG	627	6	+	Arabidopsis thaliana	cis-acting element involved in the abscisic acid responsiveness
	ABRE	ACGTG	628	5	+	Arabidopsis thaliana	cis-acting element involved in the abscisic acid responsiveness
	ABRE	CACGTG	654	6	+	Arabidopsis thaliana	cis-acting element involved in the abscisic acid responsiveness
	ABRE	ACGTG	655	5	+	Arabidopsis thaliana	cis-acting element involved in the abscisic acid responsiveness
	ABRE	CGCACGTGTC	1033	9	-	Hordeum vulgare	cis-acting element involved in the abscisic acid responsiveness
	ABRE	CACGTG	1035	6	-	Arabidopsis thaliana	cis-acting element involved in the abscisic acid responsiveness
	ABRE	ACGTG	1036	5	+	Arabidopsis thaliana	cis-acting element involved in the abscisic acid responsiveness
	ABRE	GACACGTGGC	1933	9	-	Triticum aestivum	cis-acting element involved in the abscisic acid responsiveness
	ABRE	CACGTG	1935	6	-	Arabidopsis thaliana	cis-acting element involved in the abscisic acid responsiveness
	ABRE	ACGTG	1936	5	+	Arabidopsis thaliana	cis-acting element involved in the abscisic acid responsiveness

Figure S1. ABA response elements (ABREs) in the 2000bp promoter region of *ASDs*. ABREs in 2000bp sequences upstream of the start condon of ASDs were identified by using PlantCARE (ugent.be).

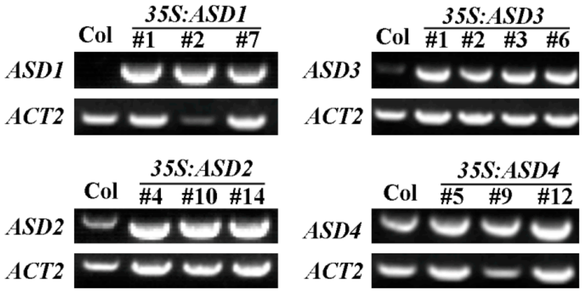

Figure S2. Expression of *ASD* genes in the overexpression transgenic plants. Total RNA was isolated from seedlings of the Col wild type and *ASD* overexpression transgenic plants, and used as template for RT-PCR analysis to examine the expression of *ASD* genes. *ACT2* was used as a control.

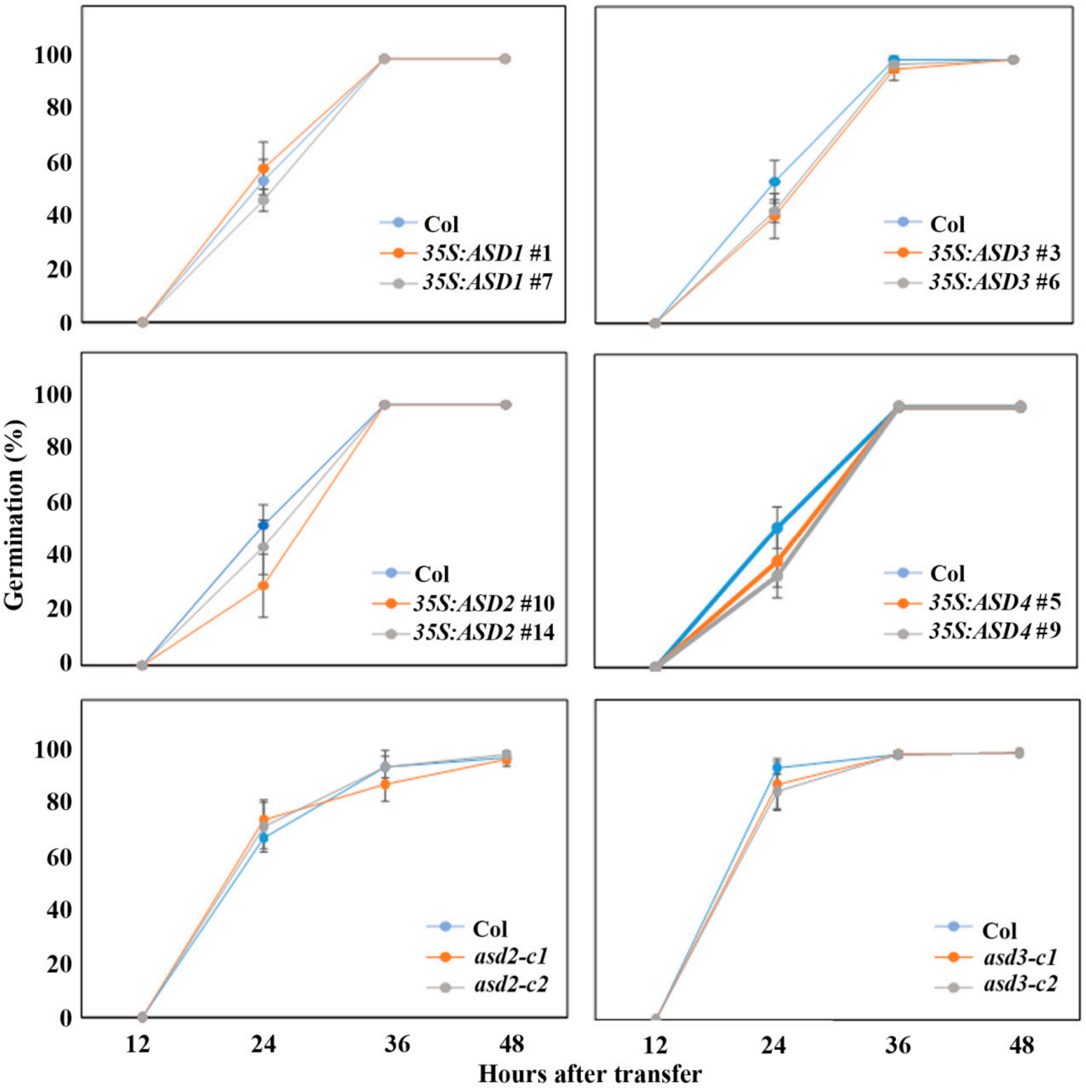

Figure S3. Seed germination of the Col wild type, the *35S:ASD* transgenic plants and single mutants *asd2* and *asd3* in control plates as indicated in Figure 4 and Figure 7A respectively.

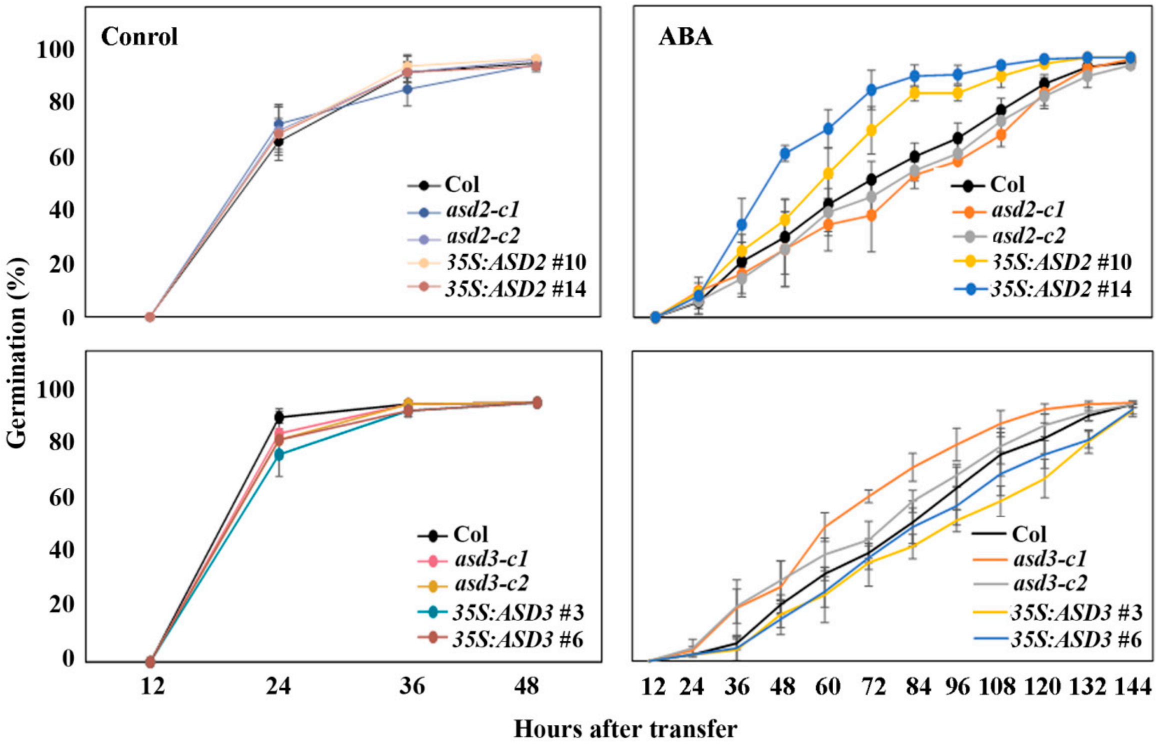

Figure S4. Effects of ABA on seed germination of the Col wild type, the *35S:ASD2* and *35S:ASD3* transgenic plants and the *asd2* and *asd3* mutants. The experiments were performed as indicated in Figure 4 and Figure 7A except that 2 μM ABA was used for the experiments.

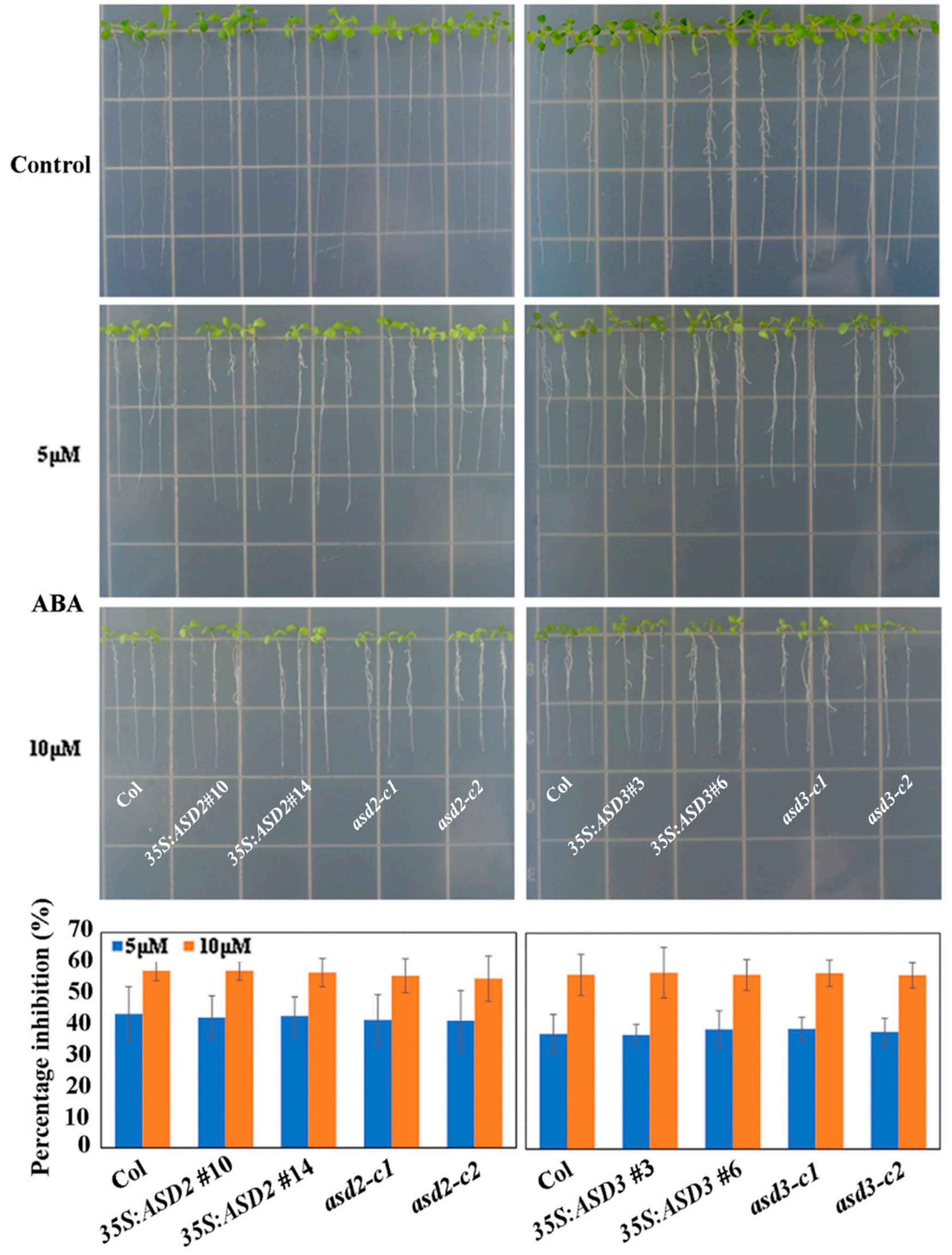

Figure S5. Effects of ABA on root elongation of the Col wild type, the *35S:ASD2* and *35S:ASD3* transgenic plants and the *asd2* and *asd3* mutants. Sterilized seeds were sown on 1/2 MS plates kept at 4 ℃ in darkness for 2 days and then transferred to a growth room grow vertically for 3 days, then seedlings were transferred into plates with or without ABA, and grow vertically for 5 day. Root length were measured before and 5 days after the transferred, percentage inhibition were calculated. Data represent the mean ± SD of 12-25 seedlings.
